# Supplementary material for: Portable Wireless Intelligent Electrochemical Sensor for the Ultrasensitive Detection of Rutin Using Functionalized Black Phosphorene Nanocomposite
Source: Molecules. 2022 Oct 5;27(19):6603. doi: 10.3390/molecules27196603 (PMC9571638; doi:10.3390/molecules27196603)
Supplement: Supplementary file 1 [file molecules-27-06603-s001.zip › molecules-1931747-supplementary.pdf]

Supplementary Information

Contents:

Figure S1. Oxidation peak currents (I_{pa}, red) and I_{pa}/I_{pc} ratio (blue) on different electrodes for 20.0 μmol L^{-1} rutin (scan rate: 0.1 V s^{-1}).

Figure S2. (a) Plots of I - υ, and (b) plots of E - lnυ of 20.0 μmol L^{-1} rutin on NF/AuNPs/N-CPDs@FLBP/CILE at different scan rate (0.03, 0.05, 0.08, 0.1, 0.2, 0.3, 0.4, 0.5, 0.6, 0.7 and 0.8 V s^{-1}).

Figure S3. The oxidation peak current of (a) repeatability and (b) reproducibility for detection of 10.0 μmol L^{-1} rutin.

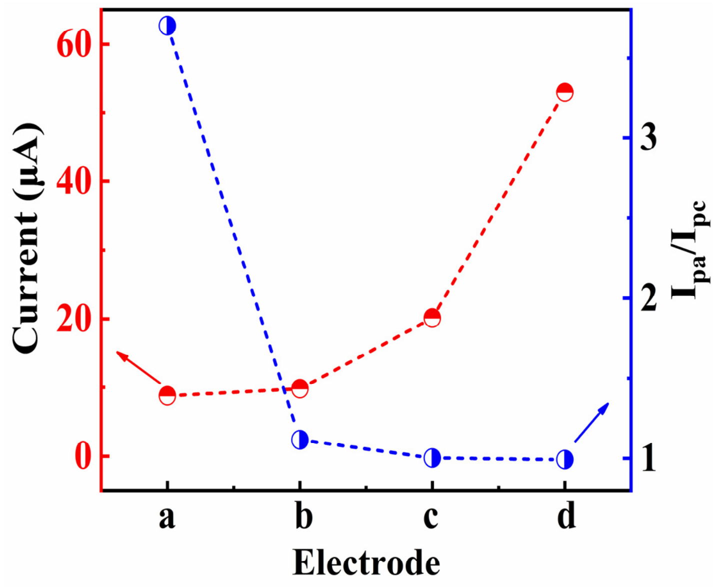

Figure S1. Oxidation peak currents (I_{pa}, red) and I_{pa}/I_{pc} ratio (blue) on different electrodes for 20.0 μmol L^{-1} rutin (scan rate: 0.1 V s^{-1}).

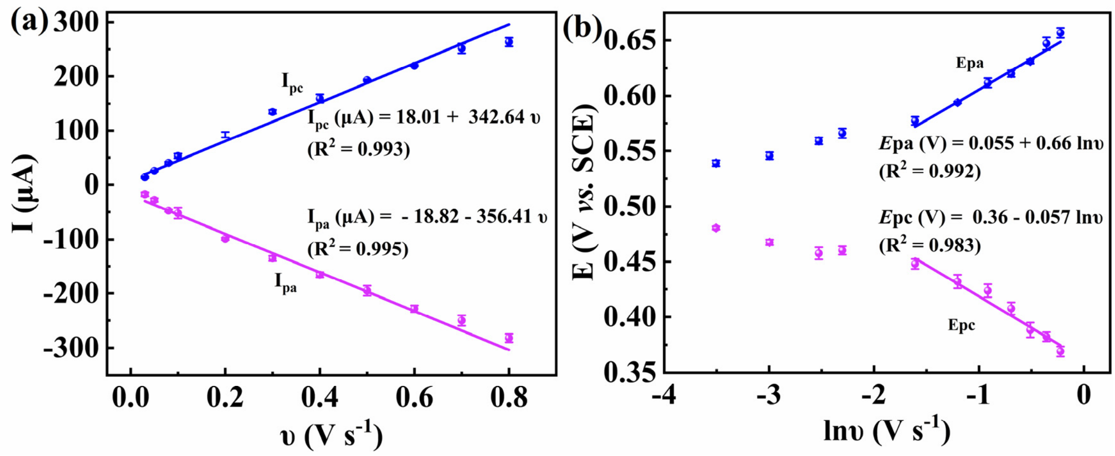

Figure S2. (a) Plots of I - υ, and (b) plots of E - lnυ of 20.0 μmol L^{-1} rutin on NF/AuNPs/N-CPDs@FLBP/CILE at different scan rate (0.03, 0.05, 0.08, 0.1, 0.2, 0.3, 0.4, 0.5, 0.6, 0.7 and 0.8 V s^{-1}).

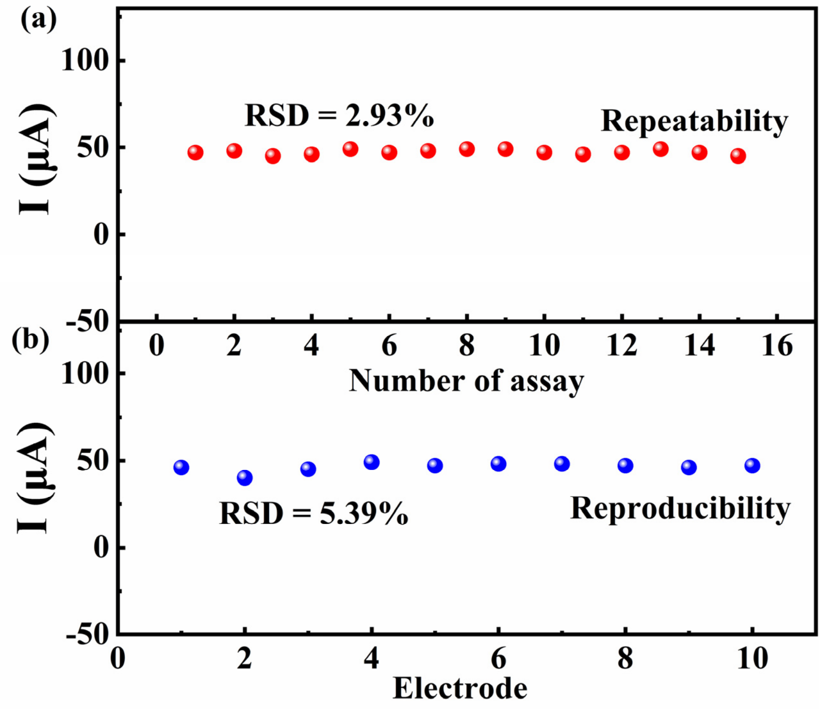

Figure S3. The oxidation peak current of (a) repeatability and (b) reproducibility for detection of 10.0 μmol L^{-1} rutin.
